# Technical approaches of 3D reconstruction from protein complex using the mixture of differently stained images: providing suggestive evidence for improving its resolution

**DOI:** 10.1186/s42649-025-00111-9

**Published:** 2025-05-28

**Authors:** Yoon Ho Park, Myeong Seon Jeong, Gang San Song, Tak gwonbaek, Young Kwan Kim, Hyun Suk Jung

**Affiliations:** 1 Department of Biochemistry, College of Natural Sciences, Kangwon National University, Chuncheon, 24341 Republic of Korea; 2 Center for Bio-Imaging Translational Research, Korea Basic Science Institute, Cheongju, 28119 Republic of Korea; 3 Department of Electrical and Electronic Engineering, College of Engineering, Kangwon National University, Chuncheon, 24341 Korea; 4 Kangwon Center for Systems Imaging, Kangwondaehak-gil, Chuncheon-si, Gangwon-do 24341 Republic of Korea

**Keywords:** 3D reconstruction, Protein complex, Negative staining, Transmission electron microscopy, Resolution

## Abstract

Negative staining electron microscopy remains a valuable tool for structural biology, particularly for initial characterization of large protein complexes. However, the limitations of single staining methods often result in incomplete structural information. Here, we present a novel multi-stain approach for negative staining electron microscopy, applied to the structural analysis of the pyruvate dehydrogenase E2 (PDH E2) complex. By integrating data from three distinct staining agents (uranyl acetate, ammonium phosphotungstate, and ammonium molybdate) we demonstrate significant improvements in structural resolution and detail. Our method improved the resolution from a range of approximately 27–31 Å (observed with individual stains) to about 21.7 Å in the combined dataset. This enhancement facilitated a clearer visualization of the complex’s icosahedral symmetry and allowed for a more precise determination of the overall shape and domain organization of the PDH E2 complex. The multi-stain approach revealed complementary structural information, with each stain highlighting different aspects of the protein complex. Uranyl acetate provided excellent overall contrast, while ammonium phosphotungstate and molybdate offered enhanced visibility of specific structural elements. The integration of these complementary data sets resulted in a more comprehensive structural model. Our findings suggest that this multi-stain negative staining approach can be a powerful tool for enhancing low-resolution structural information of large protein complexes, bridging the gap between initial characterization and high-resolution studies. This method holds promise for improving our understanding of challenging macromolecular assemblies and may serve as a valuable precursor to more advanced structural biology techniques.

**Supplementary Information:**

The online version contains supplementary material available at 10.1186/s42649-025-00111-9.

## Introduction

Negative staining electron microscopy has been a cornerstone technique in structural biology for decades, offering a rapid and accessible method for visualizing protein structures (Gallagher et al. 2019). This approach involves embedding biological specimens in a thin layer of heavy metal salts, creating a contrast-enhancing electron-dense background that outlines the specimen’s topography (Brenner and Horne 1959). Despite its widespread use, negative staining has limitations, including potential staining artifacts and the risk of sample flattening (Harris 2007). Nevertheless, it remains a valuable tool for providing initial structural information, especially for large protein complexes (Rames et al. 2014).

Large, multi-subunit protein complexes present unique challenges and opportunities for structural analysis. These complexes often possess intricate architectures with diverse structural elements, making them ideal candidates for exploring multi-stain approaches. The varying interactions between different stains and complex protein surfaces can reveal complementary structural information, potentially uncovering features that might be missed with a single staining method (Scarff et al. 2018). The size and complexity of these assemblies result in a range of surface characteristics and charge distributions, allowing for a more comprehensive evaluation of protein topographies through different staining techniques.

The choice of heavy metal stain significantly influences image quality and interpretation in negative staining electron microscopy. Commonly used stains such as uranyl acetate (UA), ammonium phosphotungstate (PTA), and ammonium molybdate (AM) each offer unique advantages (Ohi et al. 2004; Mörgelin 2017; Gunkel et al. 2024). UA provides high contrast and fine grain, excelling in overall shape visualization but potentially causing structural deformations (Booth et al. 2011). PTA offers good contrast and highlights hydrophilic regions, while AM is known for its gentler interactions, preserving delicate structures (De Carlo and Harris 2011). These stains interact differently with protein surfaces due to their charge characteristics: UA, being positively charged, binds to negatively charged regions, while PTA and AM, being negatively charged, highlight positively charged areas (Harris 2007).

The differential interactions of various stains with protein surfaces can reveal distinct structural features, potentially providing complementary information about protein architecture (De Carlo and Harris 2011). This variability in stain-protein interactions suggests that a multi-stain approach could offer a more comprehensive view of protein structure, particularly for complex macromolecular assemblies. By leveraging the strengths of different staining methods, researchers may uncover structural details that might be overlooked when using a single stain, thus enhancing our understanding of protein structure and function.

In this study, we present a novel multi-stain negative staining approach for the structural analysis of large protein complexes, using the PDH E2 complex as a model system. By systematically applying and comparing UA, PTA, and AM staining methods, we aim to extract comprehensive structural information that may not be apparent from a single staining technique. This approach not only provides more detailed insights into complex protein structures but also demonstrates the potential of combining traditional techniques in innovative ways to maximize structural information, particularly for challenging protein assemblies.

## Results

### Structural characteristics of PDH E2 complex

**Fig. 1:**
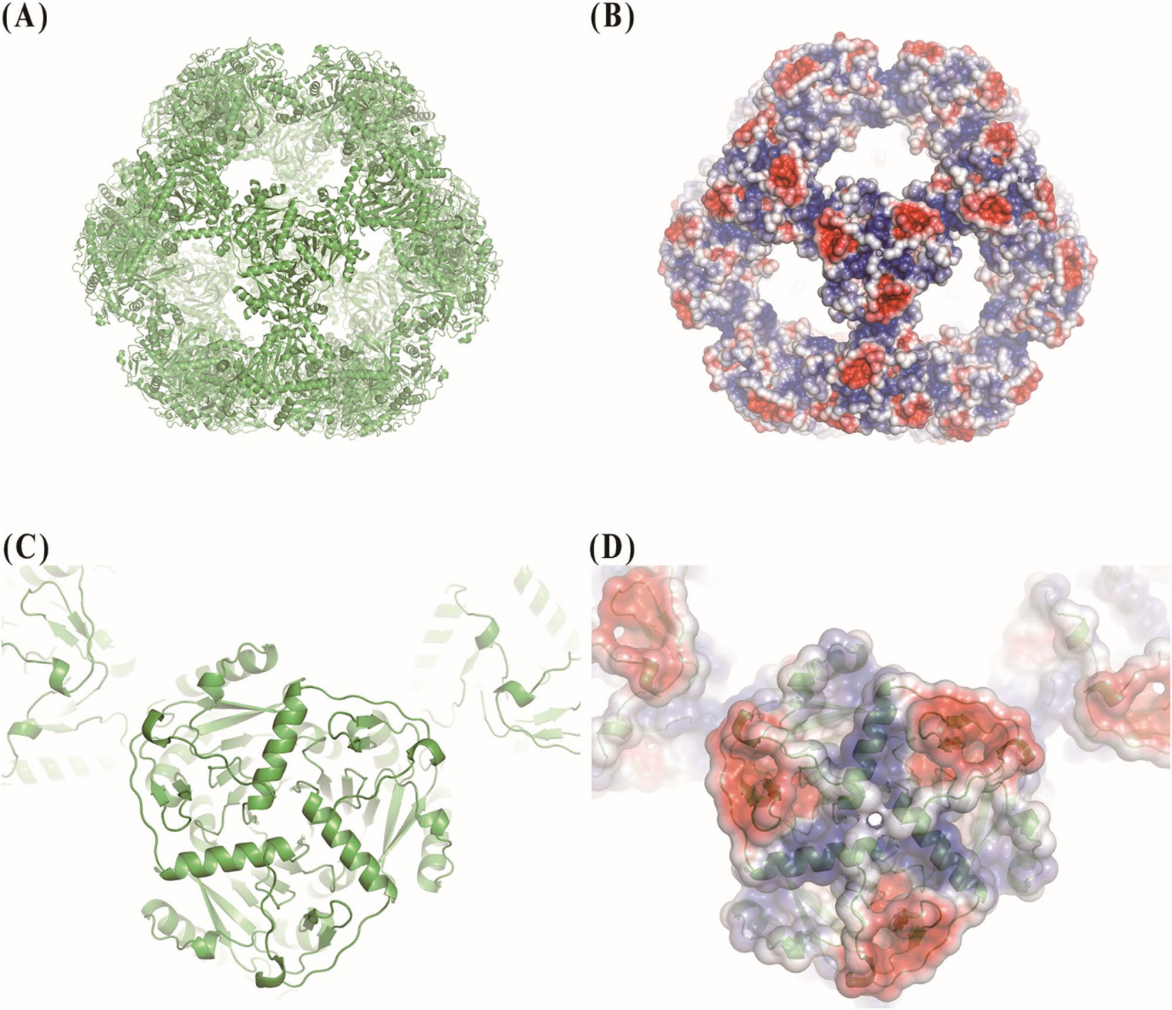
Structure and surface charge distribution of the pyruvate dehydrogenase E2 complex. (**A**) Atomic structure of the 60-mer PDH E2 complex (PDB ID: 1b5s) (Izard et al. 1999) shown in ribbon representation with each monomer colored in green. (**B**) Electrostatic surface potential of the entire complex, visualized using the APBS Electrostatics plugin in PyMOL. (**C**) Single trimeric unit in ribbon representation, highlighting the fundamental building block of the complex. (**D**) Electrostatic surface potential of a trimeric unit. In (**B**) and (**D**), blue indicates positive charge, red negative charge, and white neutral areas

The structural analysis of the PDH E2 complex from Bacillus stearothermophilus revealed a highly organized architecture with distinct charge distribution patterns (Fig. 1) (Izard et al. 1999). The complex forms a roughly spherical structure with an approximate diameter of 25–30 nm, composed of 20 trimeric units arranged in an icosahedral symmetry (Fig. 1A). Each trimer serves as a fundamental building block of the complex, with individual monomers clearly visible within the trimeric structure (Fig. 1C).

Surface charge analysis of the PDH E2 complex unveiled a striking electrostatic landscape crucial for its function and assembly (Fig. 1B, D). Notably, the regions between trimers exhibit strong positive charges, visualized as intense blue areas in the electrostatic surface representation. This positive charge distribution likely plays a key role in stabilizing the interactions between adjacent trimers, contributing to the overall integrity of the 60-mer complex. Within each trimeric unit, a distinct and repetitive pattern of charge distribution is observed. The surface of each trimer displays alternating patches of positive (blue) and negative (red) charges, forming a mosaic-like arrangement. This repetitive pattern is consistent across all visible trimers, suggesting a conserved electrostatic feature important for the complex’s function. The presence of both positively and negatively charged regions on the trimer surface may facilitate interactions with various substrates and other components of the PDH machinery, potentially guiding the efficient channeling of intermediates during the catalytic process. Furthermore, the complex’s ability to interact differentially with various heavy metal stains (UA, PTA, AM) corroborates the heterogeneous nature of its surface charge distribution, providing valuable insights into the electrostatic properties that govern its molecular interactions and catalytic efficiency.

### Comparison of negative staining methods for PDH E2 complex visualization

**Fig. 2:**
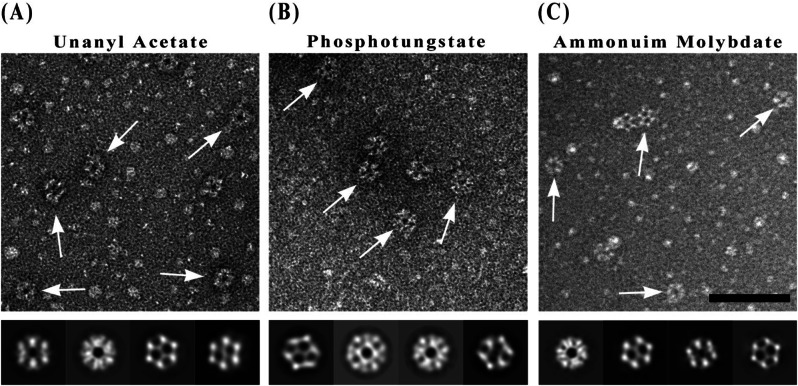
Transmission electron microscopy (TEM) analysis of PDH E2 complex using different negative stains. (**A**) Uranyl acetate, (**B**) Ammonium phosphotungstate, (**C**) Ammonium molybdate. Upper panels: Representative TEM micrographs with white arrows indicating individual PDH E2 complex particles. Lower panels: Selected 2D class averages derived from multiple aligned particles in similar orientations. These class averages reveal the characteristic features of the complex under different staining conditions and demonstrate how each stain highlights different structural aspects of the complex. Scale bar in micrographs: 100 nm

The PDH E2 complex was visualized using three different negative staining methods: UA, PTA and AM (Fig. 2). Each staining method revealed distinct structural features of the complex, providing complementary information about its architecture.

UA staining (Fig. 2A) provided high contrast and fine structural detail, clearly delineating the overall shape of the PDH E2 complex. The 2D class averages showed well-defined internal features, with distinct subunit arrangements visible within the complex. PTA staining (Fig. 2B) resulted in a slightly lower contrast compared to UA but offered improved preservation of the complex’s native structure. The 2D class averages revealed subtle structural details, particularly at the periphery of the complex, suggesting this stain may be less prone to causing structural deformations. AM staining (Fig. 2C) produced images with softer contrast but excellent structural preservation. The 2D class averages showed clear subunit organization and highlighted the complex’s symmetrical nature. This staining method appeared to be particularly effective in maintaining the integrity of more delicate structural features.

**Fig. 3:**
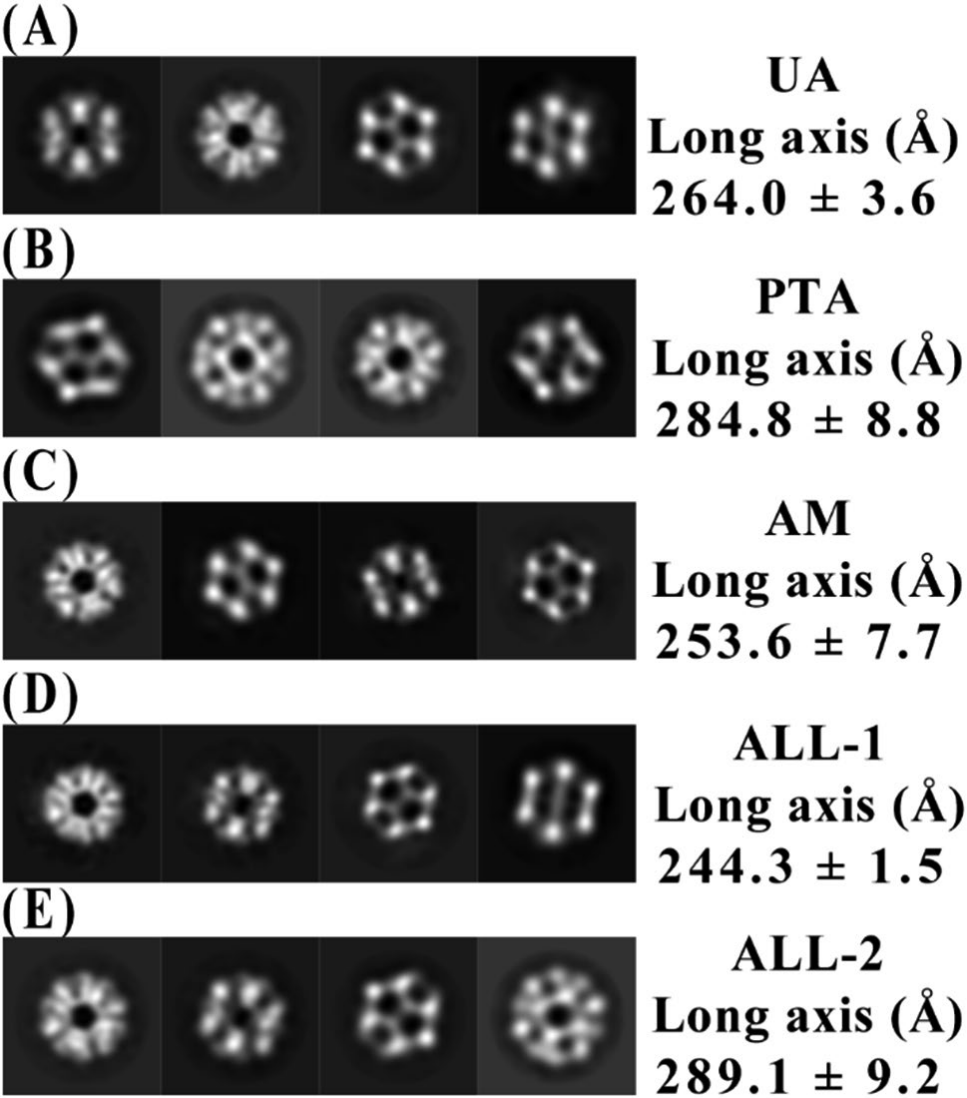
Comparison of 2D class averages and long axis measurements of PDH E2 complex under different staining conditions. (**A**) Uranyl acetate (UA), (**B**) Ammonium phosphotungstate (PTA), (**C**) Ammonium molybdate (AM), (**D**-**E**) Combined dataset (ALL) showing two distinct size classes. Each row displays representative 2D class averages selected from the final classification. Long axis measurements (mean ± SD in Ångstroms) are provided for each condition and were calculated from multiple class averages. In the combined dataset, two distinct size populations emerged: a smaller population (ALL-1, panel **D**) and a larger population (ALL-2, panel **E**). This size heterogeneity suggests effective integration of particles from different staining methods. For comparison between these size-varying classes, the first three classes in each size group were measured, revealing consistent size differences that correlate with observations from individual staining methods

Negative staining electron microscopy using three different heavy metal solutions revealed variations in the apparent size of the PDH E2 complex (Fig. 3). The long axis measurements of 2D class averages showed distinct differences: UA staining resulted in particles with an average long axis of 264.0 ± 3.6 Å (Fig. 3A), PTA staining produced larger particle images (284.8 ± 8.8 Å, Fig. 3B), while AM yielded the smallest particle images (253.6 ± 7.7 Å, Fig. 3C).

When all staining methods were combined into a single dataset (ALL), two distinct size populations emerged: a smaller population (ALL-1) with an average long axis of 244.3 ± 1.5 Å (Fig. 3D) and a larger population (ALL-2) with an average long axis of 289.1 ± 9.2 Å (Fig. 3E). Notably, the 2D class averages from the combined dataset exhibited features highly similar to those observed in the individual staining methods. Classes resembling UA, PTA, and AM stained particles were all identifiable within the ALL dataset, suggesting that the combined approach captures the structural information from each staining method.

The observed size heterogeneity among the differently stained samples (264.0 ± 3.6 Å for UA, 284.8 ± 8.8 Å for PTA, and 253.6 ± 7.7 Å for AM) can be explained by the distinct physicochemical properties of each staining agent and their specific interactions with the PDH E2 complex. Uranyl acetate, being positively charged, preferentially binds to negatively charged regions on the protein surface, creating a moderately thick layer around the complex that closely approximates the true dimensions from the crystal structure (approximately 250–260 Å). In contrast, ammonium phosphotungstate, which carries multiple negative charges, is electrostatically attracted to positively charged patches on the complex surface (particularly in the regions between trimers as shown in Fig. 1B). This stronger interaction likely creates a more substantial stain envelope around the complex, resulting in the largest apparent dimensions (284.8 ± 8.8 Å). Ammonium molybdate, also negatively charged but with different ionic radius and coordination properties compared to PTA, shows the smallest apparent size (253.6 ± 7.7 Å). This could be attributed to its greater penetration into the complex’s cavities rather than accumulating primarily on the outer surface, enabling better visualization of internal features while minimizing the external stain layer thickness. When comparing these dimensions to the crystal structure measurements, we observe that UA provides the closest match to the native structure, while PTA and AM represent slight overestimation and underestimation, respectively. Interestingly, our 2D classification analysis reveals that UA staining yields particles with dimensions most closely resembling the actual size of the complex, while the combined dataset (ALL) exhibits two distinct size populations. However, in the 3D reconstruction (Supplementary Fig. 2), both the ALL and UA maps show similar overall dimensions when visualized at the same threshold.

These stain-specific features preserved in the combined dataset offer significant advantages for structural analysis of the PDH E2 complex. These variations likely reflect differences in heavy metal binding patterns rather than conformational changes, as the E2 complex is inherently rigid. The diverse staining interactions, including internal and external binding of heavy metals, provide a more comprehensive representation of the complex’s structure. This approach enhances low-resolution structural details and aids in more precise particle classification during 3D reconstruction. By leveraging complementary information from multiple staining methods, we can obtain a more complete understanding of the PDH E2 complex’s structure, particularly in the crucial low-resolution range that defines its overall shape and organization. This multi-stain strategy maximizes the structural information obtainable through negative stain electron microscopy, potentially revealing features that might be missed with a single staining method.

### Resolution analysis and structural features

**Fig. 4:**
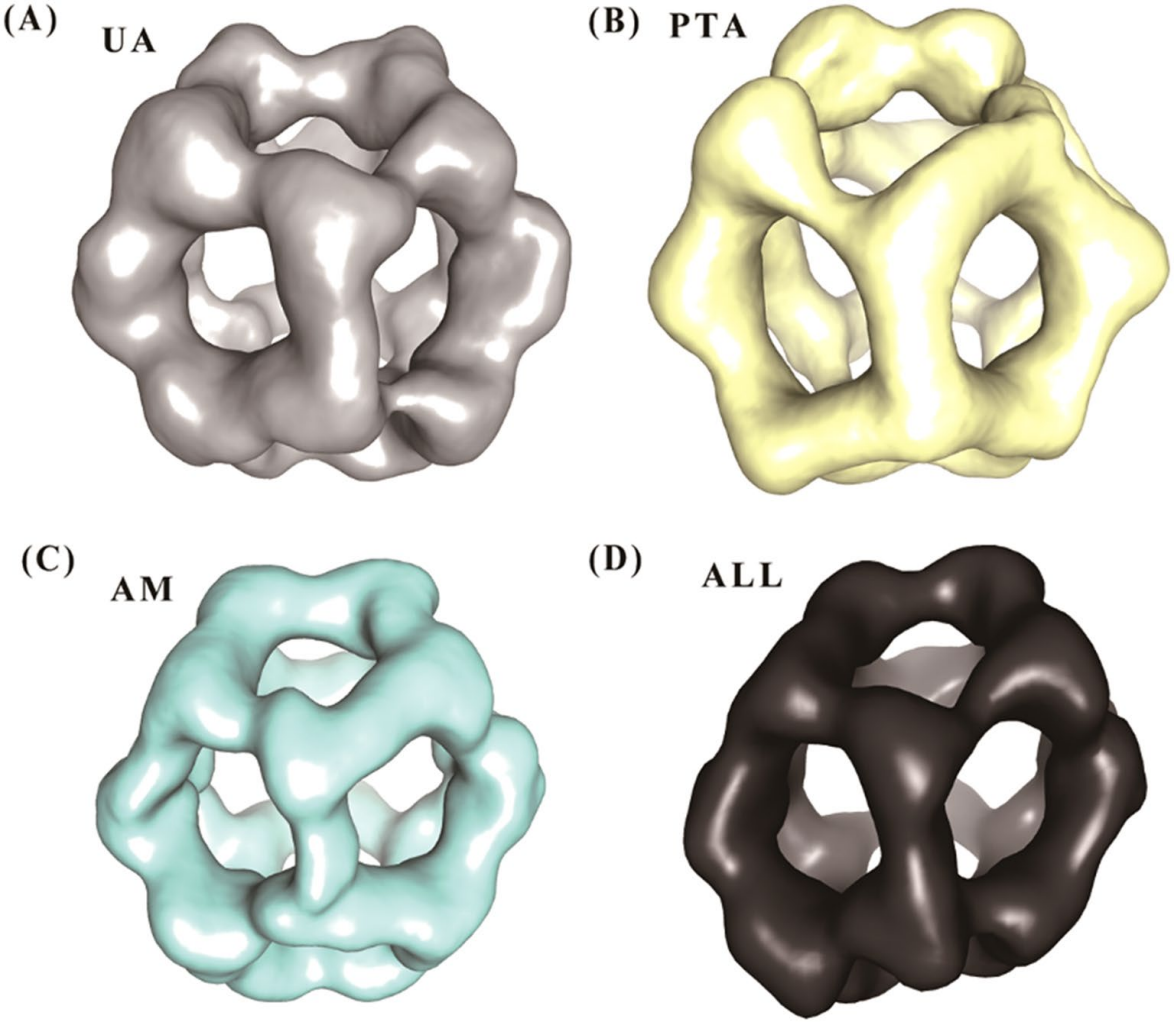
Resolution analysis of PDH E2 complex reconstructions using different staining methods. (**A**) Uranyl acetate (UA), (**B**) Ammonium phosphotungstate (PTA), (**C**) Ammonium molybdate (AM), and (**D**) Combined dataset (ALL). Each panel shows the 3D reconstruction visualized at the same threshold level (0.03) and in the same orientation to facilitate direct comparison

The resolution analysis of the PDH E2 complex reconstructions obtained from different staining methods revealed distinct structural features and resolution capabilities (Fig. 4).

The multi-stain approach for the structural analysis of the PDH E2 complex demonstrated significant advantages in both resolution and structural detail compared to individual staining methods. Fourier Shell Correlation (FSC) analysis revealed varying resolutions for each staining technique (Fig. 4). The UA staining yielded a resolution of 31.4 Å, while PTA and AM staining resulted in resolutions of 30.3 Å and 27.2 Å, respectively, based on the FSC 0.143 criterion. Notably, the combined dataset integrating all three staining methods (ALL) achieved a markedly improved resolution of 21.7 Å.

The enhanced resolution obtained from the multi-stain approach translated into improved visualization of structural features. The combined reconstruction exhibited better definition of the complex’s icosahedral symmetry, with clearer delineation of individual trimeric units. This improvement was particularly evident when comparing the combined reconstruction to those from single staining methods. The multi-stain approach provided complementary information, effectively leveraging the strengths of each staining method to produce a more comprehensive representation of the PDH E2 complex structure.

Quantitatively, the combined method resulted in a 20.2% improvement in resolution compared to the best single-stain method (AM), and a 30.9% improvement over the commonly used UA staining. These results underscore the effectiveness of the multi-stain approach in enhancing the structural information obtainable through negative staining electron microscopy for the PDH E2 complex.

### Local resolution analysis of PDH E2 complex reconstructions

**Fig. 5:**
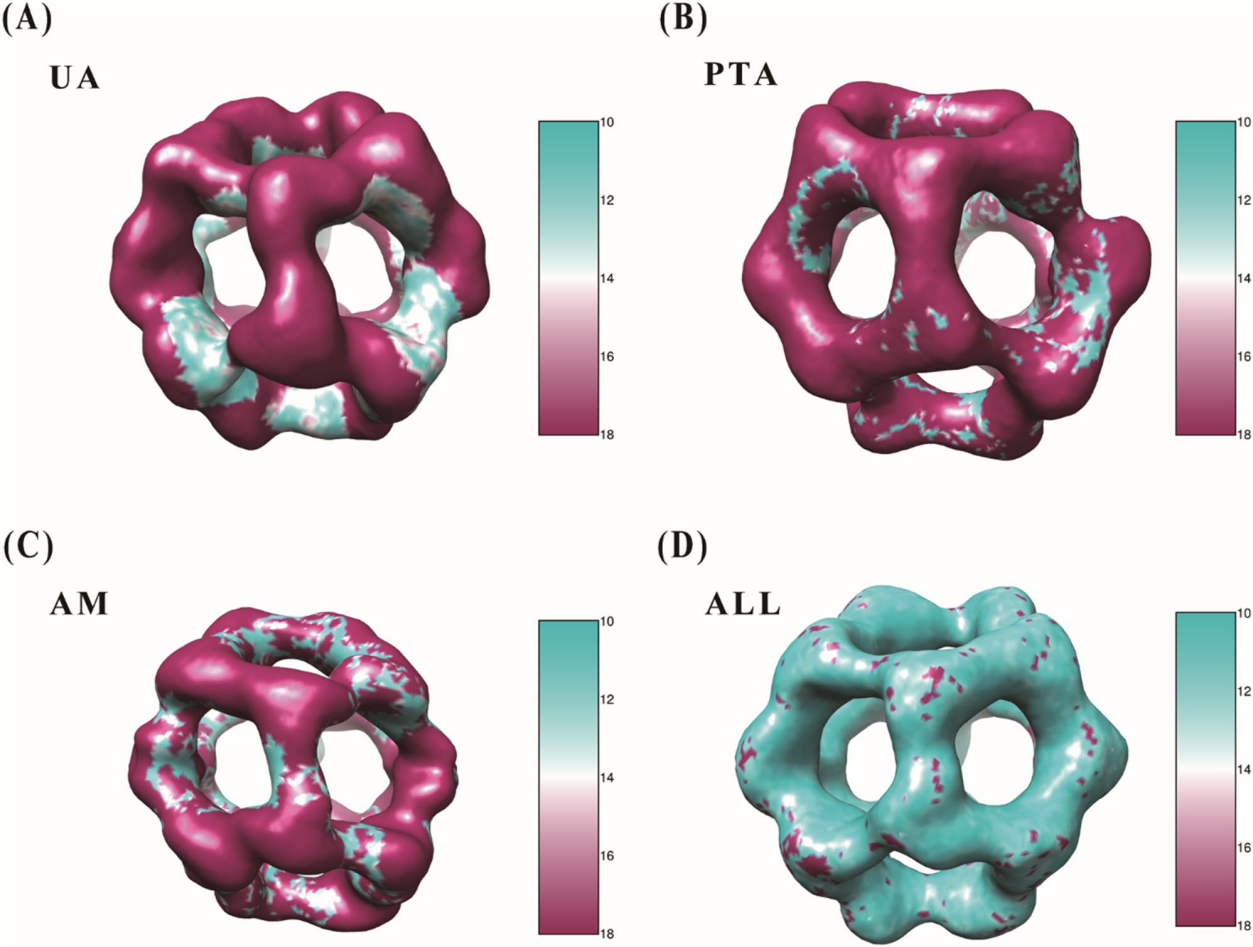
Local resolution analysis of PDH E2 complex reconstructions. Local resolution maps of PDH E2 complex reconstructions obtained from different staining methods: (**A**) Uranyl acetate (UA), (**B**) Ammonium phosphotungstate (PTA), (**C**) Ammonium molybdate (AM), and (**D**) Combined dataset (ALL). All EM maps are displayed at the same contour level of 0.03 to ensure direct comparability. The color scale represents local resolution values ranging from 10 Å (cyan) to 18 Å (magenta). Note the predominance of cyan coloration in the combined dataset (**D**), indicating higher overall resolution throughout the complex compared to single staining methods. Each staining method shows distinctive patterns of local resolution, with certain regions better resolved in different stains, demonstrating the complementary nature of the multi-stain approach. Areas of higher resolution (cyan) are particularly visible in the inner cavities and along structural interfaces

To gain deeper insights into the structural details of the PDH E2 complex, we performed local resolution analysis using RELION’s implementation of ResMap and compared the resolution values obtained from different staining conditions (Fig. 5) (Kucukelbir et al. 2014).

The UA staining reconstruction exhibits predominantly magenta coloration (14–18 Å resolution), with cyan areas (10–12 Å resolution) visible in the inner cavities (Fig. 5A). The PTA reconstruction displays magenta regions with cyan patches distributed along the inner edges of the openings (Fig. 5B). The AM reconstruction shows a distribution of magenta regions (14–18 Å) with cyan patches (10–12 Å) along the inner surfaces (Fig. 5C).

The combined multi-stain approach (ALL) reconstruction is predominantly cyan colored, indicating higher overall resolution (10–12 Å) throughout the complex (Fig. 5D). The local resolution analysis demonstrates that the combined dataset provides improved resolution compared to any single staining method.

These local resolution maps provide valuable information about the structural details preserved by each staining method. While the absolute values differ between the two methods, the relative trends remain consistent, with the multi-stain approach demonstrating superior resolution and detail across the complex.

## Discussion

In this study, we have demonstrated the efficacy of a multi-stain negative staining approach for the structural analysis of the PDH E2 complex. By integrating data from three distinct staining agents (UA, PTA, and AM) we achieved a significant improvement in resolution and structural detail compared to individual staining methods. The combined dataset yielded a resolution of 21.7 Å, surpassing the resolutions obtained from single stains (ranging from 27.2 Å to 31.4 Å), and provided a clearer visualization of the icosahedral symmetry of the complex.

The success of this multi-stain approach stems from the complementary nature of the information provided by each stain. UA, a positively charged stain, binds preferentially to negatively charged regions of the protein surface, offering high contrast and clear delineation of the overall shape. Conversely, negatively charged stains like PTA and AM highlight positively charged areas, revealing additional structural features. Given the PDH E2 complex’s heterogeneous surface charge distribution characterized by distinct positive and negative patches as shown in Fig. 1 this variability in stain-protein interactions enabled a more comprehensive depiction of its architecture. By combining these datasets, we effectively mitigated limitations inherent to single-stain methods, such as incomplete visualization due to selective stain binding.

It is crucial to contextualize the resolution values reported here within the limitations of negative staining electron microscopy. Unlike Cryo-EM, where resolution serves as a robust indicator of structural detail and is typically assessed using the FSC at a 0.143 threshold, the concept of resolution in negative staining reconstructions has different implications. Negative staining relies on heavy metal salts to outline the protein’s surface, producing 2D projections rather than capturing internal structural details. Consequently, the resolutions achieved (21.7 Å for the combined dataset and 27.2–31.4 Å for individual stains) are typical for this technique but do not approach the near-atomic precision attainable with cryo-EM. The improvement from 31.4 Å (UA) to 21.7 Å (combined dataset) is notable within the scope of negative staining, yet it reflects an enhancement in surface detail rather than a fundamental leap in structural resolution. Thus, while the multi-stain approach refines the low-resolution structural model, resolution metrics must be interpreted appropriately within this context.

Further insight into the structural benefits of this approach emerges from comparing local resolution and FSC-based resolution. Local resolution analysis, conducted using ResMap-1.1.4, revealed variations across the PDH E2 complex, with the combined multi-stain reconstruction exhibiting higher resolution (10–12 Å in some regions) compared to single-stain maps (14–18 Å predominantly). This enhancement suggests that integrating multiple stains averages out artifacts and inconsistencies introduced by individual stains, yielding a more uniform and detailed reconstruction. In contrast, the FSC-based resolution of 21.7 Å for the combined dataset represents an overall average across the entire 3D model. The discrepancy arises because local resolution highlights specific areas of improved detail while FSC reflects a global measure influenced by factors such as particle alignment and stain penetration variability. For the PDH E2 complex, the multi-stain approach thus provides a more consistent structural representation, particularly valuable for a complex with diverse surface properties.

## Conclusion

In conclusion, our study introduces a novel multi-stain negative staining approach that enhances the structural analysis of the PDH E2 complex, offering a more detailed and comprehensive view than traditional single-stain methods. By integrating data from UA, PTA and AM, we leveraged their complementary staining properties to achieve improved resolution and a clearer depiction of the complex’s architecture. This method proved particularly effective for the PDH E2 complex, given its heterogeneous surface charge distribution, highlighting its potential as a tool for initial structural characterization of large protein assemblies.

Nevertheless, these findings must be approached with appropriate caution, recognizing the inherent limitations of negative staining. This technique remains a low-resolution method, unable to rival the detailed insights provided by advanced approaches like Cryo-EM. Moreover, the effectiveness of the multi-stain strategy may vary depending on the specific surface properties and structural stability of the protein complex under study. For complexes lacking the charge diversity or rigidity of large protein assemblies, benefits might be less pronounced.

Despite these constraints, the multi-stain approach offers a practical and accessible means to obtain a more accurate initial model of complex protein structures. For the PDH E2 complex, it has provided valuable insights into its overall shape and organization, serving as a foundation for subsequent high-resolution investigations. While not a replacement for advanced techniques, this method represents a promising option for structural biologists seeking to refine low-resolution models in the early stages of analysis, particularly for challenging macromolecular assemblies.

Looking forward, this multi-stain negative staining approach could be especially valuable for analyzing diverse structural questions in macromolecular biology. It could be applied to study conformational changes in large protein complexes under different functional states, characterize the architecture of membrane protein assemblies, and provide foundation models for integrative structural biology approaches. The method may also be particularly useful for size-based measurements of large protein assemblies, quantitative assessment of structural heterogeneity, and preliminary characterization of protein-protein interaction interfaces before proceeding to higher-resolution techniques. By extracting more comprehensive low-resolution structural information, this approach bridges the critical gap between initial sample characterization and detailed structural analysis, potentially accelerating our understanding of complex macromolecular machines that underpin cellular functions.

## Methods

### PDH E2 complex analysis and visualization

The PDH E2 complex from Bacillus stearothermophilus was purified following the method described (Kim et al. 2024a). Structural data were obtained from the Protein Data Bank (PDB) using the entry 1B5S (Izard et al. 1999). Surface charge distribution analysis was conducted using the APBS Electrostatics plugin in PyMOL (Schrödinger, LLC) to predict and visualize the electrostatic potential and charge regions of the protein complex. This allowed us to assess the spatial distribution of positive and negative charges across the protein’s surface. Protein structure and surface visualizations were performed using PyMOL, while electron microscopy map visualizations were carried out using UCSF Chimera.

### Negative staining

Purified PDH E2 complex samples were subjected to negative staining using three different heavy metal stains: uranyl acetate, ammonium phosphotungstate, and ammonium molybdate. For each staining method, 5 µL of purified PDH E2 complex (concentration: 0.05 mg/mL) was applied to a glow-discharged carbon-coated copper grid and allowed to adsorb for 30 s. Excess sample was blotted off with filter paper. The grids were then stained with 3 drops of either 1% (w/v) uranyl acetate, 1% (w/v) ammonium phosphotungstate (pH 7.0), or 1% (w/v) ammonium molybdate (pH 7.0). Excess stain was removed by blotting, and the grids were allowed to air-dry (Kim et al. 2024b, c).

### Transmission electron microscope and image processing

Micrographs were collected on a Tecnai 10 transmission electron microscope (FEI, USA) operated at 100 kV and recorded on an UltraScan 1000 CCD camera (Gatan, USA) at a magnification of 34,000×, corresponding to a pixel size of 3.2 Å. Three negative staining conditions were employed: uranyl acetate (UA), ammonium phosphotungstate (PTA), and ammonium molybdate (AM). For each staining condition, 300 micrographs were collected.

Particle picking was performed using RELION’s built-in Laplacian-of-Gaussian (LoG) autopicking algorithm with manual curation to remove false positives. After two rounds of 2D classification in RELION 5.0 to discard poor-quality classes, the remaining particles were used for 3D reconstruction. The final reconstructions utilized 2,323 particles for PTA, 2,838 particles for UA, and 2,792 particles for AM datasets, respectively. Additionally, a combined dataset (ALL) was generated by pooling data from all three staining methods, with 2,584 particles used in the final consensus reconstruction (ratio of stained particles from three staining can be evenly distributed for the reconstruction).

Initial 3D models were generated via RELION’s stochastic gradient descent (SGD) routine and subsequently refined using the 3D auto-refine procedure with C1 symmetry applied. Local resolution estimation was performed using ResMap-1.1.4 (Kucukelbir et al. 2014) for each reconstruction to guide further interpretation.

## Electronic supplementary material

Below is the link to the electronic supplementary material.

Supplementary Material 1: Figure S1. Fourier shell correlation (FSC) analysis of the PDH E2 complex reconstructions using different negative staining methods. (A) FSC curve of uranyl acetate (UA) stained sample showing a resolution of 31.4Å at the 0.143 criterion. (B) FSC curve of ammonium phosphotungstate (PTA) stained sample with a resolution of 30.3Å. (C) FSC curve of ammonium molybdate (AM) stained sample demonstrating a resolution of 27.2Å. (D) FSC curve of the combined dataset (ALL) integrating particles from all three staining methods, achieving an improved resolution of 21.7Å. All FSC curves were calculated using the EMDB FSC Calculator web service. The gold-standard FSC threshold of 0.143 was used to determine resolution estimates. Fig. S2 Comparison of 3D reconstructions with fitted atomic model. (A) Uranyl acetate (UA), (B) Ammonium phosphotungstate (PTA), (C) Ammonium molybdate (AM), (D) Combined dataset (ALL), and (E) Difference map between ALL and UA reconstructions. All EM maps are displayed at the same contour level of 0.03 with transparency set to 0.65. The atomic model of the PDH E2 complex (PDB ID: 1B5S) (Izard et al. 1999) was manually fitted into each reconstruction to assess the structural correspondence.

## Data Availability

Not applicable.

## Abbreviations

PDH: Pyruvate Dehydrogenase
EM: Electron Microscopy
TEM: Transmission Electron Microscope
UA: Uranyl Acetate
PTA: Ammonium Phosphotungstate
AM: Ammonium Molybdate
FSC: Fourier Shell Correlation
PDB: Protein Data Bank
APBS: Adaptive Poisson-Boltzmann Solver
ALL: Combined dataset (used to refer to the merged data from all three staining methods)
3D: Three-Dimensional
2D: Two-Dimensional
