# Supplementary material for: Technical approaches of 3D reconstruction from protein complex using the mixture of differently stained images: providing suggestive evidence for improving its resolution
Source: Appl Microsc. 2025 May 28;55:6. doi: 10.1186/s42649-025-00111-9 (PMC12119436; doi:10.1186/s42649-025-00111-9)
Supplement: Supplementary file 1 — Supplementary Material 1: Figure S1. Fourier shell correlation (FSC) analysis of the PDH E2 complex reconstructions using different negative staining methods. (A) FSC curve of uranyl acetate (UA) stained sample showing a resolution of 31.4Å at the 0.143 criterion. (B) FSC curve of ammonium phosphotungstate (PTA) stained sample with a resolution of 30.3Å. (C) FSC curve of ammonium molybdate (AM) stained sample demonstrating a resolution of 27.2Å. (D) FSC curve of the combined dataset (ALL) integrating particles from all three staining methods, achieving an improved resolution of 21.7Å. All FSC curves were calculated using the EMDB FSC Calculator web service. The gold-standard FSC threshold of 0.143 was used to determine resolution estimates. Fig. S2 Comparison of 3D reconstructions with fitted atomic model. (A) Uranyl acetate (UA), (B) Ammonium phosphotungstate (PTA), (C) Ammonium molybdate (AM), (D) Combined dataset (ALL), and (E) Difference map between ALL and UA reconstructions. All EM maps are displayed at the same contour level of 0.03 with transparency set to 0.65. The atomic model of the PDH E2 complex (PDB ID: 1B5S) (Izard et al. 1999) was manually fitted into each reconstruction to assess the structural correspondence. [file 42649_2025_111_MOESM1_ESM.docx]

**Figure S1. Fourier Shell Correlation (FSC) analysis of the PDH E2 complex reconstructions using different negative staining methods.** (A) FSC curve of uranyl acetate (UA) stained sample showing a resolution of 31.4Å at the 0.143 criterion. (B) FSC curve of ammonium phosphotungstate (PTA) stained sample with a resolution of 30.3Å. (C) FSC curve of ammonium molybdate (AM) stained sample demonstrating a resolution of 27.2Å. (D) FSC curve of the combined dataset (ALL) integrating particles from all three staining methods, achieving an improved resolution of 21.7Å. All FSC curves were calculated using the EMDB FSC Calculator web service. The gold-standard FSC threshold of 0.143 was used to determine resolution estimates.


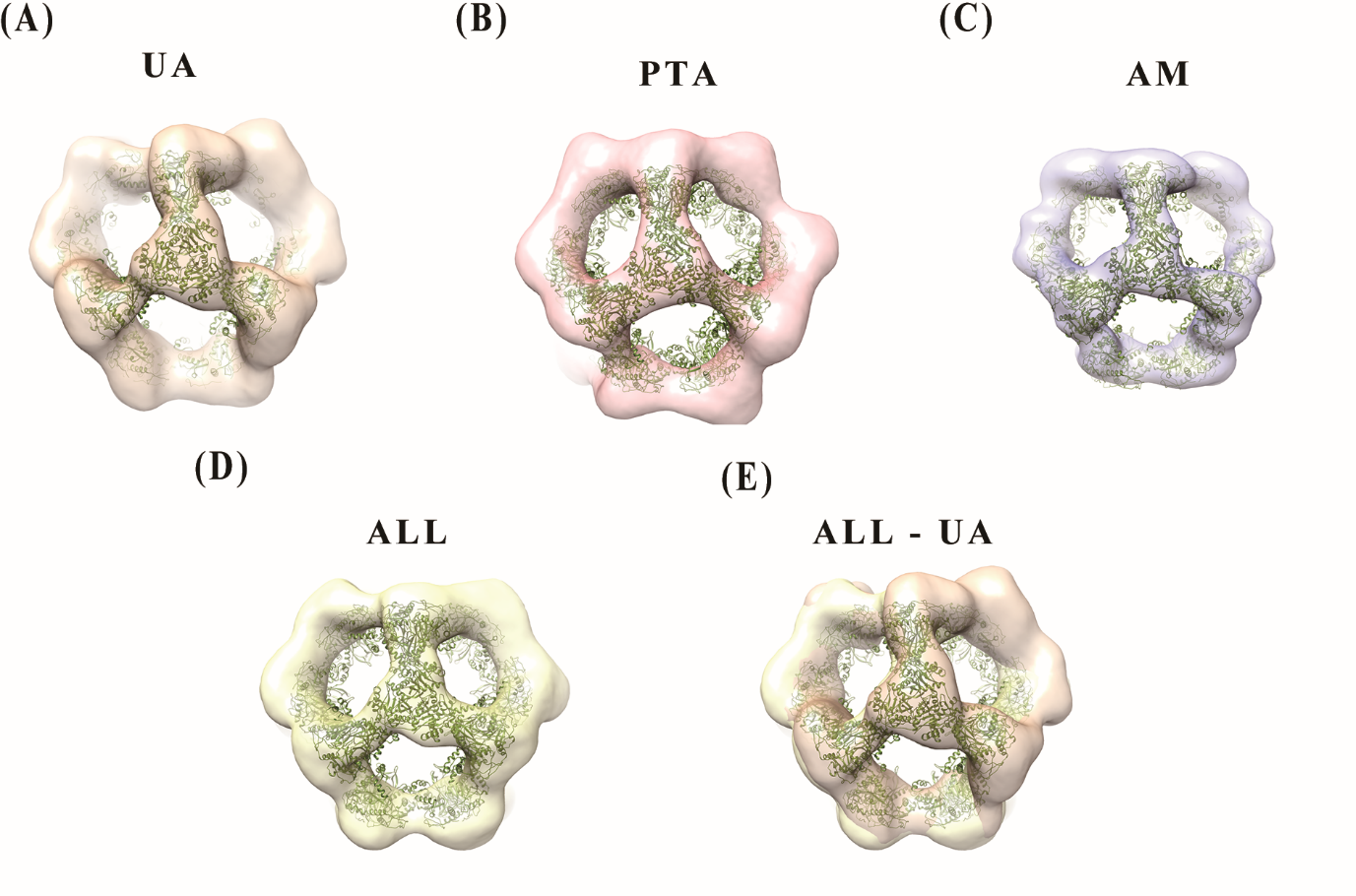


**Figure S2. Comparison of 3D reconstructions with fitted atomic model.** (A) Uranyl acetate (UA), (B) Ammonium phosphotungstate (PTA), (C) Ammonium molybdate (AM), (D) Combined dataset (ALL), and (E) Difference map between ALL and UA reconstructions. All EM maps are displayed at the same contour level of 0.03 with transparency set to 0.65. The atomic model of the PDH E2 complex (PDB ID: 1B5S) (Izard et al., 1999) was manually fitted into each reconstruction to assess the structural correspondence.
